# Atypical parathyroid adenoma: Series of two consecutive cases from a tertiary care hospital in Qatar

**DOI:** 10.1016/j.ijscr.2022.107296

**Published:** 2022-06-10

**Authors:** Mohamed S. AL Hassan, Walid El Ansari, Adham Darweesh, Mahir Petkar, Abdelrahman Abdelaal

**Affiliations:** a Department of General Surgery, Hamad General Hospital, Doha, Qatar; b Department of Surgery, Hamad General Hospital, Doha, Qatar; c College of Medicine, Qatar University, Doha, Qatar; d Weill Cornell Medicine–Qatar, Doha, Qatar; e Department of Clinical Imaging, Hamad General Hospital, Doha, Qatar; f Department of Laboratory Medicine & Pathology, Hamad Medical Corporation, Doha, Qatar

**Keywords:** Primary hyperparathyroidism, Atypical parathyroid adenoma, Hypercalcemia, SPECT scan, Parathyroidectomy

## Abstract

**Introduction:**

Atypical parathyroid adenomas (APA) are an uncommon cause of hypercalcemia and comprise a minority of parathyroid adenomas.

**Presentation of cases:**

Case 1 - Egyptian male, 48 years old with history of type 2 diabetes mellitus, incidentally discovered increased serum of calcium level on routine investigation, was diagnosed as PHPT, US and MIBI scan showed large left inferior parathyroid adenoma, focused exploration and excision of the APA was undertaken, histopathology confirmed APA. Case 2 - Egyptian male, 60 years old, cardiac patient with history of diabetes, hypertension and multiple cardiac interventions, had nausea, vomiting, constipation abdominal pain, polyuria, polydipsia, and history of passing renal stones, hypercalcemia workup showed primary hyperparathyroidism (PHPT), MIBI was negative and SPECT scan suggested right inferior parathyroid adenoma, focused exploration and excision of the APA was undertaken, histopathology confirmed APA.

**Discussion:**

APA are an uncommon cause of hypercalcemia and are responsible for a minority of parathyroid adenomas. Combined US and MIBI and SPECT scans can detect APA. Focused exploration and excision of the APA under general anaesthesia can completely remove the APA.

**Conclusion:**

Awareness of the physician and a high index of suspicion to symptoms or signs that could reflect an underlying PHPT is essential. Yearly biochemical and neck US follow up are required to detect any risk of recurrence or malignancy in the long term.

## 1 Introduction

Primary hyperparathyroidism (PHPT) is due to the presence of an adenoma/single-gland disease in 80–85 % of cases, while multiple gland disease or hyperplasia accounts for 10–15 % of cases of PHPT [1]. Atypical parathyroid adenoma (APA) and parathyroid carcinoma (PC) are both responsible for 1.2–1.3 % and ≤1 % of PHPT, respectively [1]. APA lies in the scale between adenoma and carcinoma, and is a relatively rare cause of PHPT [2].

The clinical manifestations of PC among most patients are essentially indistinguishable from those of patients with APA or a parathyroid adenoma [3]. Likewise, operative findings cannot distinguish APA from PC reliably, hence, at the time of initial surgery, differentiation from PC can be difficult [4]. In the same time, APA shares a few histological characteristics with PC [4]. Thus, APA represents a challenging diagnosis for both the surgeon and the pathologist, especially when establishing a differential diagnosis against PC [1]. Others have noted that some ambivalent tumors have previously been reported as PC, but clinically, their behaviour has not always been consistent with such diagnosis [5].

In the current retrospective single-centre case series at Hamad General Hospital (largest tertiary care hospital) in Doha, Qatar, we review two consecutive cases of APA (2016–2020). We report the two cases in line with the PROCESS criteria [6]. To the best of our knowledge, this APA case series is one of the very few globally, and is probably the first case series reported from the MENA region and Africa.

## 2 Case presentations

### 2.1 Case 1

Egyptian male, 48 years old with history of type 2 diabetes mellitus controlled by lifestyle modifications and medications was found to have persistent increased serum of calcium level on routine investigation at the outpatient clinic our institution in 2016 (Hamad Medical Corporation, the largest tertiary care facility in Qatar). Family history was unremarkable, he was a non-smoker, and was taking vitamins C and D and esomeprazole. Past surgical history was unremarkable, and the patient had no other chronic diseases. He felt well with no complaints, no abdominal pain, constipation, bone pain, or history of renal stones, or other clear symptoms or signs of hypercalcemia. Upon physical examination, he was conscious, alert and appeared to be in no acute distress. Neck examination showed a query lower left sided neck swelling. The rest of the physical examination was unremarkable. His initial laboratory workup showed HbA1c 5.7 %, Ca level of 2.94 mmol/L (adjusted Ca 2.76 mmol/L), high PTH of 237 pg/ml, albumin was 49 g/L, alkaline phosphatase 176 U/L, free T4 12.17 pmol/L, phosphorus 1.26 mmol/L, TSH 1.18 mIU/L, and uric acid 489 umol/L.

**Fig. 1:**
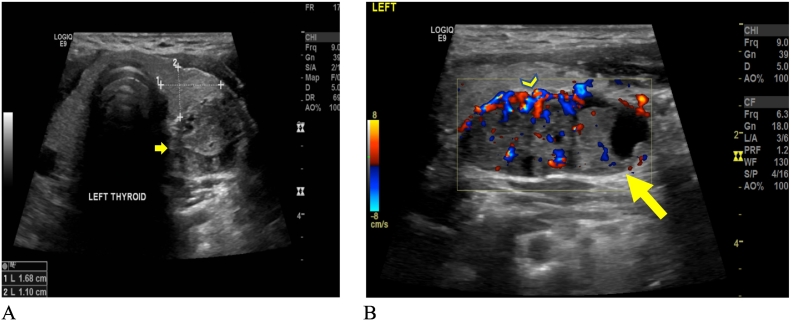
Thyroid ultrasound. Thyroid Ultrasound (A-B) showing well-defined, mildly lobulated, solid and cystic, predominantly solid, with mixed echogenicity, oval lesion (yellow arrow) with increased peripheral vascularity (arrowhead) lying posterior to the left lobe of the thyroid gland. (For interpretation of the references to color in this figure legend, the reader is referred to the web version of this article.)

**Fig. 2:**
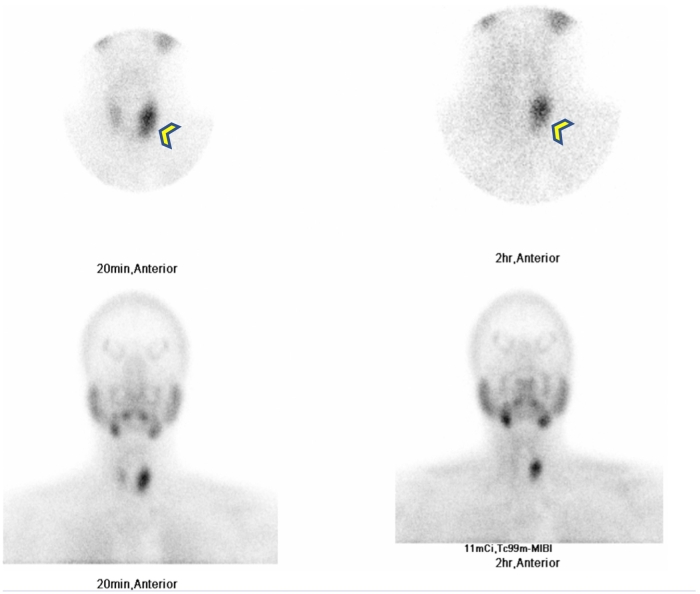
Parathyroid 99m Tc-Sestamibi scintigraphy.

The diagnostic workup included ultrasound (US) of thyroid which showed that both lobes and isthmus of thyroid gland were normal in size, parenchymal echotexture, and vascularity, with no obvious intraparenchymal focal lesions. There was evidence of a well-defined mildly lobulated mixed echogenic (solid and cystic), predominantly solid oval lesion measuring approximately 4.5 × 1.5 cm with increased peripheral vascularity lying posterior to the left lobe of the thyroid gland (Fig. 1A and B). Most parts of the lesion appeared separate from the thyroid gland, features likely suggestive of a parathyroid lesion, probably a neoplasm, and the recommendation was further evaluation. Nuclear medicine parathyroid MIBI showed evidence of a large left inferior parathyroid adenoma (Fig. 2). Early images (left) revealed tracer uptake in the thyroid tissues with a more intense uptake in a large focus related to the lower pole of the left thyroid lobe (arrowhead); Late images revealed physiological tracer washout from thyroid tissues but retained activity in the above-described focus, consistent with an active left inferior parathyroid adenoma.

**Fig. 3:**
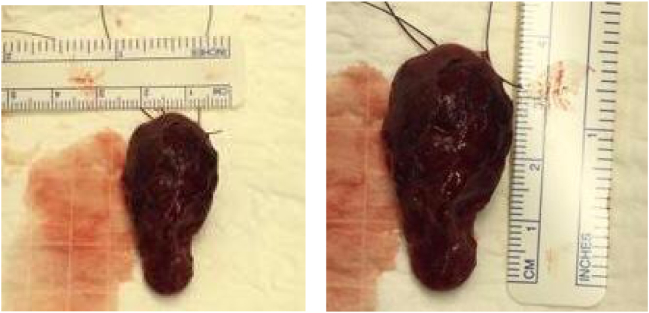
Excised PA showing its measurement.

The patient was diagnosed with PHPT due to parathyroid adenoma. He was discussed at the multidisciplinary team (MDT) meeting and it was concluded that he should undergo excision of the adenoma. The patient received no medication and was scheduled directly for surgery. In December 2016, he underwent left parathyroidectomy, and rapid PTH at 15 min after excision was 44 pg/ml. The surgery was undertaken by an experienced consultant thyroid/parathyroid surgeon. We also undertook a frozen section which confirmed that the excised specimen is parathyroid gland. Post-surgery, the patient was stable and was discharged on post-op day 1. Fig. 3 shows the excised specimen.

**Fig. 4:**
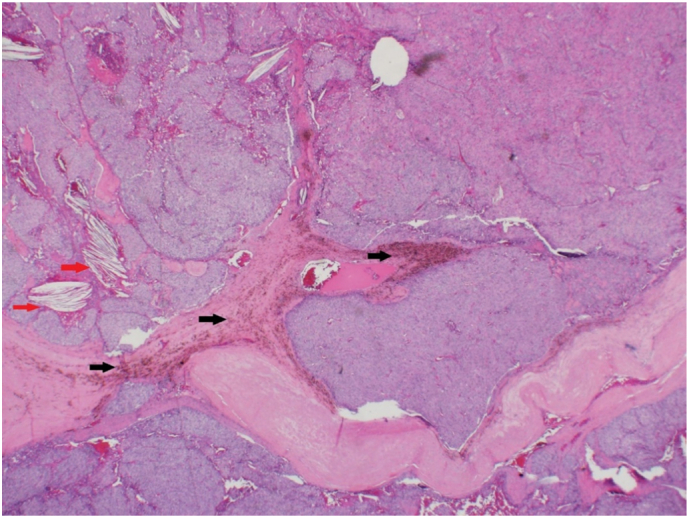
Atypical parathyroid adenoma showing parathyroid tissue separated into irregular nodule by thick fibrous bands. Note the aggregates of hemosiderin laden macrophages (black arrow) and the cholesterol clefts (red arrow). (H and E ×4). (For interpretation of the references to colour in this figure legend, the reader is referred to the web version of this article.)

The biopsy findings indicated the maximum tumor dimensions of 3.5 × 2 × 1 cm (5.9 g), with focal hemorrhage, infarction with hemosiderin laden macrophages and mitotic rate <1/10HPF. The features were worrisome of an atypical proliferation including fibrous bands as well as extension into the capsule, but with no pleomorphism or significant mitotic activity identified. There were foci where the tumor was adjacent to vessels, but no definitive diagnostic vascular invasion was noted. These features were considered to represent at least an APA (Fig. 4). The specimen was sent to a specialist centre for confirmation of the diagnosis, who concluded that the appearances were consistent with a parathyroid adenoma, with some atypical features including the presence of thick fibrous septae that divide the parathyroid tissue into regular lobules as well as a focally irregular intersection with the surrounding soft tissues. Unequivocal histologic evidence of carcinoma was not observed such as lympho-vascular or perineural invasion or frank invasion into the surrounding soft tissues. Based on the atypical features, the recommendation was follow-up of the patient with monitoring of the PTH hormone and serum Ca levels.

**Fig. 5:**
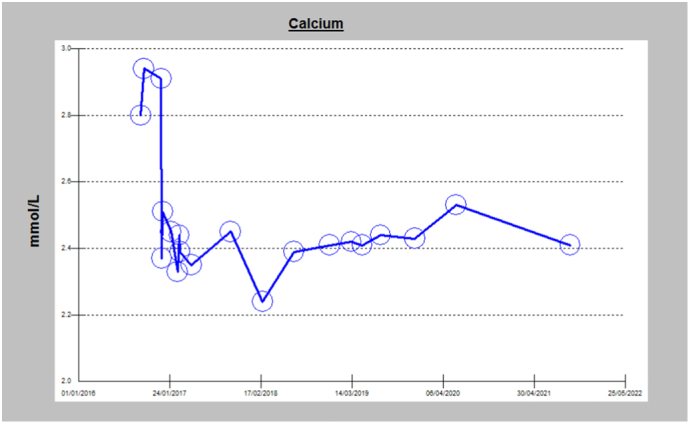
Calcium levels before and after surgery.

Follow up of this patient has been over 6 years, with no evidence of recurrence of hypercalcemia. Fig. 5 depicts the six year profile of calcium level (2016–2022) and shows the high calcium level before surgery that returned to normal after surgery, and was maintained.

### 2.2 Case 2

In August 2019, an egyptian male, 60 years old, with history of diabetes, hypertension and percutaneous coronary intervention, was admitted with myocardial infarction to the heart hospital at our institution. He underwent percutaneous coronary intervention that subsided his pain. He had no family history of thyroid/parathyroid disease, but was a current smoker. He was known to have high hypercalcemia (corrected calcium 3.25 mmol/L) since about 1.5 years earlier, but parathyroid MIBI scan at that time showed no evidence of parathyroid adenomas. Medications for the hypercalcemia were prescribed, he was discharged, but was subsequently lost to follow up.

**Fig. 6:**
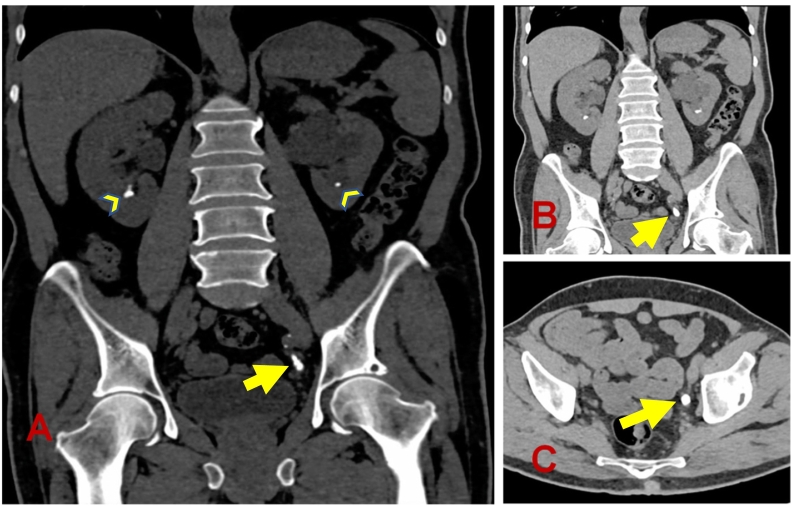
Plain CT urinary tract. Multiple left distal ureteric calculi (A-C, yellow arrows) with moderate to severe left hydroureteronephrosis. Multiple bilateral renal calculi are also noted (arrowheads). (For interpretation of the references to color in this figure legend, the reader is referred to the web version of this article.)

In December 2021, the patient came to the emergency room of our hospital with dyspnea at rest and orthopnea, and was diagnosed as acute decompensated heart failure. In this index admission, he reported incomplete compliance with the hypercalcemia medications that were prescribed for him. He had history of nausea and vomiting, constipation and abdominal pain in the last month. In addition, he experienced polyuria, polydipsia, and history of passing renal stones. There was no bone pain, and no history of fractures. He had persistent elevated adjusted calcium and PTH (Ca highest level 3.55 mmol/L; PTH highest level 839 pg/ml). The patient was medically treated for hypercalcemia with hydration, calcitonin and zoledronate. CT scan of the urinary tract in order to check for renal or ureteric stones (Fig. 6) showed multiple lower left ureteric calculi, (largest 10 × 6 mm), with associated moderate to severe left hydroureteronephrosis, no significant perinephric stranding, multiple bilateral non-obstructive renal calculi (largest about 7 mm), bilateral adrenal nodular lesions (largest on the right about 18 × 17 mm).

**Fig. 7:**
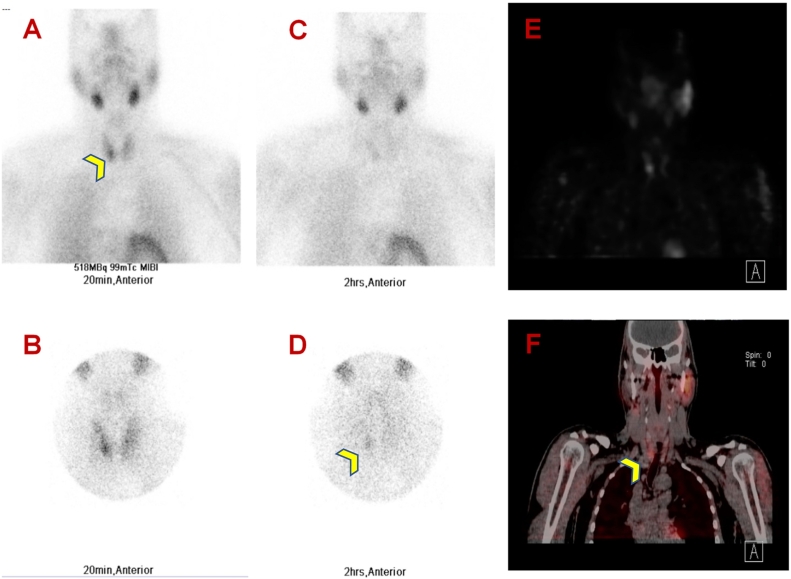
Parathyroid 99m Tc-sestamibi scintigraphy SPECT/CT. Early images (A-B) revealed tracer uptake in the thyroid tissues with a more intense uptake in a small focus related to the lower pole of right thyroid lobe (arrowhead); Late images (C-D) revealed physiological tracer washout from thyroid tissues but retained activity in the above-described focus; small focal radiotracer uptake (E-F) at the right lower pole of the thyroid gland, consistent with an active right inferior parathyroid adenoma.

Nuclear medicine parathyroid SPECT (single proton emission computerized tomography) scan after intravenous injection of technetium 99 MIBI (Fig. 7) showed small, abnormal uptake at the right lower pole of the thyroid gland, findings suggestive of a small right inferior parathyroid adenoma.

He was scheduled for and went for excision of the parathyroid adenoma. PTH was 142 and 110 pg/ml after 10 and 20 min of the excision respectively. We also undertook a frozen section which confirmed that the excised specimen is parathyroid gland. On postoperative day 1, the patient was stable, not in distress with clean wound, calcium was 2.64 mmol/L (adjusted 2.79 mmol/L) and PTH was 13 pg/ml. The patient was discharged.

**Fig. 8:**
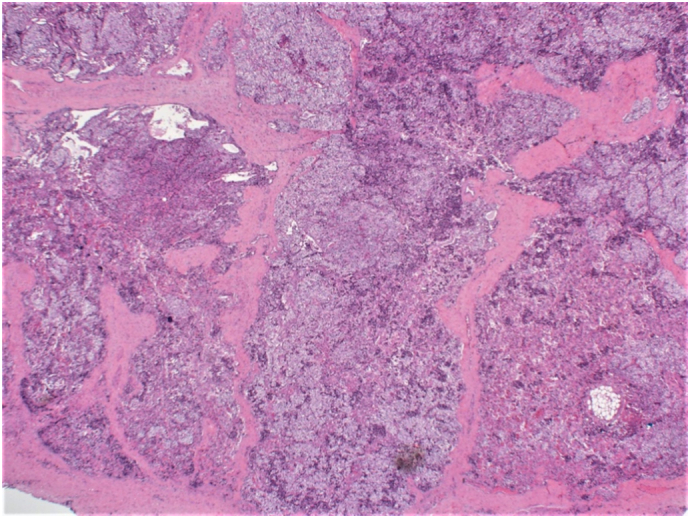
Variably thickened fibrous septa, dividing the parathyroid tissue into lobules (H and E ×4).

The final histopathology result showed atypical parathyroid adenoma (Fig. 8) with maximum tumor dimensions 2.5 × 1.2 × 1 cm (3.5 g). As with Case 1, the slides were sent to a specialist center for confirmation of the diagnosis. Their report indicated that the histologic sections revealed the presence of parathyroid tissue with focal mild cytologic atypia, divided into regular lobules by fibrous septa of varying thickness. There was no unequivocal evidence of perineural invasion. There was a focus where parathyroid tissue was present within the lumen of a vessel, but this was believed to represent an artifact rather than true lympho-vascular invasion. In addition, the Ki-67 revealed a low proliferation index. The decision was that the histologic finding would be most in keeping with an atypical parathyroid adenoma. Their recommendation was close follow-up with monitoring of the PTH and serum Ca levels.

**Fig. 9:**
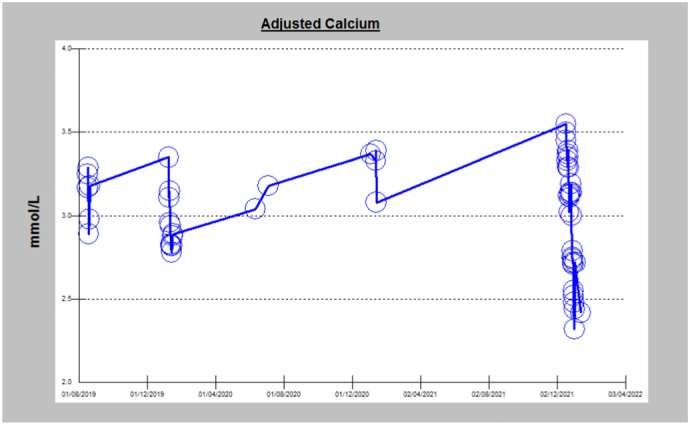
Calcium levels before and after surgery.

Follow up of this patient is in progress. Nevertheless, his calcium profile from 2019 to 2022 shows a drop to normal levels as depicted in Fig. 9.

## 3 Discussion

APA is a term used to describe a neoplasm with ‘worrisome’ features but not accomplishing the ‘absolute histopathological criteria of malignancy’ [2]. We describe two cases of this rare adenoma. To the best of our knowledge, this report is the first from the MENA region to describe such cases. Two APA were reported from Turkey [7].

In terms of PHPT caused by the APA, our second patient had complaints suggestive of PHPT (nausea, vomiting, constipation, abdominal pain, polyuria, polydipsia, history of passing renal stones), and was recommended for MIBI scan which returned negative results. However, our first case felt well, had no complaints, no abdominal pain, constipation, bone pain, or history of renal stones, or other symptoms or signs of hypercalcemia, and was diagnosed after the incidental findings of PHPT on routine investigation for type 2 diabetes mellitus. Hence, these two cases highlight the fact that PHPT can be easily missed; or might present in an atypical fashion, with the patient feeling well with no clear symptoms or signs of hypercalcemia.

Hence, awareness of the physician and maintaining a high index of suspicion to symptoms or signs that could reflect an underlying covert PHPT is essential. Only after the diagnosis of hypercalcemia can the reason for it be investigated, as illustrated in the current two case studies. This is important, as PHPT is frequently due to an adenoma/single-gland disease in 80–85 % of cases [1]. In Italy, among 117 PHPT patients, histological and immunohistochemical assessment found that 10 patients (8.5 %) had ATA [1].

As for demography, both our APA patients were males aged 48 and 60 years. In connection with sex, others found a 30 % male proportion among 117 PHPT patients [1]. For age, although a study reported a mean age of 60 ± 13 years [1], the age range is wide, where a 75-year-old female with ectopic APA has been described [8], while another report depicted a 16-year-old adolescent girl who presented with severe PHPT manifestations due to APA [2]. Nevertheless, patients with an inherited condition causing APA present much younger, often in third decade [9].

In terms of investigation, for our second patient, we undertook parathyroid MIBI and SPECT scans, where the former showed no evidence of PA, while the latter suggested small right inferior PA. For our first case, we conducted US and MIBI, and the former showed a predominantly solid oval lesion lying posterior to the left lobe of the thyroid gland, and the latter showed evidence of a large left PA. A positive parathyroid sestamibi scan, if correctly interpreted and applied, truly represents a parathyroid adenoma, never a “false-positive” scan [10]. We agree with others that conventional imaging methods (US and 99mTc-MIBI scintigraphy) are successful in up to 90 % of cases, especially when combined, while negative/inconclusive results occurred in remaining patients [8]. Similarly, others used 4-dimensionsal computed tomography for localization of the PA, and Technetium 99 SESTAMIBI scan that confirmed the location of the adenomas in the same region without any ectopic lesion [2]. The SPECT scan we undertook for the second case also agrees with reports that the excellent diagnostic performance of radio-labelled choline PET/CT as well as its low radiation burden, renders this method valuable to substitute other current imaging procedures [8], including US, MIBI scintigraphy, 4D CT, and MRI and results in a rapid and reliable “one-stop-shop” preoperative localization of hyperfunctioning parathyroids [11].

Histologically, APAs are parathyroid tumors that show features worrisome for parathyroid carcinoma, but lack any diagnostic features for parathyroid carcinoma such as unequivocal invasive growth, vascular/perineural invasion, soft tissue, or surrounding structures (including thyroid, recurrent laryngeal nerve, trachea, esophagus) invasion or documented metastatic disease, according to WHO criteria for malignancy. The worrisome features include clinical/intraoperative adherence and presence of thick capsule, pseudocapsular invasion, bands of fibrosis (with or without associated hemosiderin deposition), pronounced trabecular growth, nuclear atypia, prominent nucleoli, and mitotic activity (>1/10 high-power fields (HPFs)) [1]. Both our cases were sampled extensively to identify any invasive features, but there was no conclusive histological evidence of malignancy in either case.

In terms of surgery, for both our cases, we undertook focused exploration and excision of the APA under general anaesthesia. Others described a four-gland neck exploration and excision of the right lower parathyroid gland for a patient with ATA [9]. However, still others reported an ATA in a young adolescent girl with poor respiratory reserve, where en bloc resection of the right parathyroid with right sided hemithyroidectomy was undertaken using local anaesthesia [2]. For both our cases, we conducted rapid intra-operative parathyroid hormone (PTH) during surgery that showed a sharp drop in PTH level that satisfied the Miami Criterion (>50 % PTH drop from either the greatest pre-incision or pre-excision PTH measurement in a blood sample 10 min after complete resection of a hyperfunctioning gland) for successful parathyroid gland resection, in line with others [9]. In addition, intraoperatively, we also undertook a frozen section which confirmed that the excised specimen is parathyroid gland for both cases. However, radioguided parathyroid operations employing a gamma probe to assess the physiologic activity of any/all of the parathyroid glands have been described [12], as measures of sequestered radioactivity is a very accurate appraisal of individual parathyroid gland hormone production [13]. APA and parathyroid cancer share some histological characteristics, hence at the time of initial surgery, differentiation can be challenging, and operative findings cannot discriminate APA from parathyroid cancer reliably [4].

As for follow up, there is a risk of recurrence or malignancy in the long term [2]. Whilst follow up of our second case is in progress as surgery was since only a few months, follow up of our first case over 6 years confirmed that the high calcium level before surgery returned to normal after surgery and was maintained suggesting no recurrence of APA. Long-term studies found a 3 % recurrence of APA [14]. Thus, yearly biochemical appraisal and neck ultrasound after surgery has been recommended in order to assess any recurrence [15].

This case series is limited by the number of cases, however, patients with APA are rare. Despite this, the case series has strengths including that the slides from both cases were sent to a specialist center for confirmation of the diagnosis and the diagnoses was confirmed in both cases.

## 4 Conclusion

APA are an uncommon cause of hypercalcemia and are responsible for a minority of parathyroid adenomas. Awareness of the physician and a high index of suspicion to symptoms or signs that could reflect an underlying PHPT is essential. Combined US and MIBI and SPECT scans can detect APA. Focused exploration and excision of the APA under general anaesthesia can completely remove the APA. Yearly biochemical and neck US follow up are required to detect any risk of recurrence or malignancy in the long term.

## Registration of research studies

Not first in Man.

## Sources of funding

Nothing to declare.

## Ethical approval

Approved by Medical Research Center, Hamad Medical Corporation reference number (MRC-04-22-266).

## Consent

Written informed consent was obtained from the patients for publication of this case series and accompanying images. This is available for the Editor-in-Chief of this journal on request.

## Provenance and peer review

Not commissioned, externally peer-reviewed.

## Author contribution

Mohamed S. Al Hassan: study concept, data interpretation, editing and reviewing the paper. Walid El Ansari: study concept, data interpretation, writing the paper. Adham Darweesh: ultrasonographic data interpretation, reviewing the paper. Mahir Petkar: pathology data interpretation, reviewing the paper. Abdelrahman Abdelaal: study concept, data interpretation, reviewing the paper. All authors read and approved the final version.

## Guarantor

Prof Dr. Walid El Ansari: welansari9@gmail.com.

## Declaration of competing interest

Nothing to declare.

## References

[1] Galani A., Morandi R., Dimko M., Molfino S., Baronchelli C., Lai S., Gheza F., Cappelli C., Casella C. (2021). Atypical parathyroid adenoma: clinical and anatomical pathologic features. World J. Surg. Oncol..

[2] Boro H., Alam S., Kubihal V., Khatiwada S., Kubihal S., Agarwal S., Khadgawat R. (2022). Atypical parathyroid adenoma: severe manifestations in an adolescent girl. Pediatr. Endocrinol. Diabetes Metab..

[3] Levin K.E., Galante M., Clark O.H. (1987). Parathyroid carcinoma versus parathyroid adenoma in patients with profound hypercalcemia. Surgery.

[4] Ippolito G., Palazzo F.F., Sebag F., De Micco C., Henry J.F. (2007). Intraoperative diagnosis and treatment of parathyroid cancer and atypical parathyroid adenoma. Br. J. Surg..

[5] Sandelin K., Auer G., Bondeson L., Grimelius L., Farnebo L.O. (1992). Prognostic factors in parathyroid cancer: a review of 95 cases. World J. Surg..

[6] Agha R.A., Sohrabi C., Mathew G., Franchi T., Kerwan A., N. O'Neill for the PROCESS Group (2020). The PROCESS 2020 guideline: updating consensus Preferred Reporting Of CasE Series in Surgery (PROCESS) guidelines. Int. J. Surg..

[7] Demıralay E., Altaca G., Demırhan B. (2011). Morphological evaluation of parathyroid adenomas and immunohistochemical analysis of PCNA and Ki-67 proliferation markers. Turk Patoloji Derg..

[8] Knappe L., Paone G., Giovanella L. (2021). An ectopic, dysmorphic and atypical parathyroid adenoma. Endocrine.

[9] Newman C., Costello M., Casey M., Davern R., Dinneen K., Lowery A., McHale T., O'Shea P.M., Quinn A.M., Bell M. (2021 Jul). A case of adrenal Cushing's syndrome and primary hyperparathyroidism due to an atypical parathyroid adenoma. Ther. Adv. Endocrinol. Metab..

[10] Norman J.G., Jaffray C.E., Chheda H. (2000). The false-positive parathyroid sestamibi: a real or perceived problem and a case for radioguided parathyroidectomy. Ann. Surg..

[11] Giovanella L., Bacigalupo L., Treglia L.G., Piccardo A. (2021). Will 18F-choline PET/CT replace other methods of preoperative parathyroid imaging?. Endocrine.

[12] Norman J., Politz D. (2009). 5,000 parathyroid operations without frozen section or PTH assays: measuring individual parathyroid gland hormone production in real time. Ann. Surg. Oncol..

[13] Norman J., Politz D. (2008). Measuring individual parathyroid gland hormone production in real-time during radioguided parathyroidectomy. Experience in over 8,000 operations. Minerva Endocrinol..

[14] Cetani F., Marcocci C., Torregrossa L., Pardi E. (2019). Atypical parathyroid adenomas: challenging lesions in the differential diagnosis of endocrine tumors. Endocr. Relat. Cancer.

[15] Cardoso L., Stevenson M., Thakker R.V. (2017). Molecular genetics of syndromic and non-syndromic forms of parathyroid carcinoma. Hum. Mutat..

